# Comparison of Hypervirulent and Non-Hypervirulent Carbapenem-Resistant *Acinetobacter baumannii* Isolated from Bloodstream Infections: Mortality, Potential Virulence Factors, and Combination Therapy In Vitro

**DOI:** 10.3390/antibiotics13090807

**Published:** 2024-08-26

**Authors:** Likang Yao, Ningjing Liu, Yingyi Guo, Chuyue Zhuo, Xu Yang, Yijing Wang, Jiong Wang, Feifeng Li, Jiahui Li, Nanhao He, Jiakang Chen, Yexin Lin, Shunian Xiao, Chao Zhuo

**Affiliations:** State Key Laboratory of Respiratory Disease, The First Affiliated Hospital of Guangzhou Medical University, Guangzhou 510000, China

**Keywords:** *Acinetobacter baumannii*, bloodstream infection, carbapenem-resistant, virulence, checkerboard dilution

## Abstract

Hypervirulent carbapenem-resistant *Acinetobacter baumannii* (hv-CRAB) has emerged in bloodstream infections (BSI). Cases of BSI caused by hv-CRAB (hv-CRAB-BSI) had posed a significant threat to hospitalized patients. In this study, 31 CRAB strains isolated from Chinese BSI patients were analyzed, of which 24 were identified as hv-CRAB-BSI and 7 as non-hv-CRAB-BSI, using the *Galleria mellonella* infection model. Patients with hv-CRAB-BSI had higher rates of septic shock (79.2% vs. 14.3%, *p* = 0.004) and mortality (66.7% vs. 14.3%, *p* = 0.028). All strains were resistant to most antibiotics but sensitive to colistin. Hv-CRAB-BSI showed lower resistance to minocycline than non-hv-CRAB-BSI (54.2% vs. 100%, *p* = 0.03). Whole-genome sequencing revealed that the detection rates of immune modulation genes *ptk* and *epsA* in hv-CRAB-BSI were significantly higher than in non-hv-CRAB-BSI (91.7% vs. 28.6%, *p* = 0.002). Additionally, all ST457 hv-CRAB-BSI lacked *abaR*, and all ST1486 non-hv-CRAB-BSI lacked *adeG*. The checkerboard dilution method assessed the efficacies of various antibiotic combinations, revealing that although synergism was rarely observed, the combination of colistin and minocycline showed the best efficacy for treating CRAB-BSI, regardless of whether the infections were hv-CRAB-BSI or non-hv-CRAB-BSI. These findings highlight the importance of analyzing molecular characteristics and exploring effective treatment strategies for hv-CRAB-BSI.

## 1. Introduction

*Acinetobacter baumannii* is the primary pathogen responsible for bloodstream infections (BSI) in hospitalized patients [1,2]. According to the “Global Burden of Diseases, Injuries, and Risk Factors Study (GBD) 2019 Antimicrobial Resistance Collaborators” report, BSI caused by *Acinetobacter baumannii* accounted for a significant number of deaths, resulting in 247,000 deaths globally in 2019 [3].

The resistance of *Acinetobacter baumannii* is a factor influencing the prognosis of patients. Carbapenem antibiotics are primary agents used for the treatment of *Acinetobacter baumannii* infections [4]. However, as reported by the China Antimicrobial Resistance Surveillance Network (CHINET) program (http://www.chinets.com/), the resistance rates of *Acinetobacter baumannii* to imipenem and meropenem increased from 32.9% and 41.3% in 2005 to 71.2% and 71.9% in 2022, respectively. Currently, treatment options for carbapenem-resistant *Acinetobacter baumannii* (CRAB) are limited. Therefore, CRAB was classified as a critical-priority bacterium for the research and development of novel antibiotics by the World Health Organization (WHO) in 2017 [5]. Furthermore, CRAB has been associated with the prognosis of patients with BSI. In 2015, a single-center study in China reported that the mortality rate of patients with BSI caused by CRAB (66.7%) was higher than that of patients with carbapenem-sensitive *Acinetobacter baumannii* (CSAB) (23.1%) [6]. However, several studies have indicated that poor outcomes in patients with carbapenem-resistant *Acinetobacter baumannii* bloodstream infections (CRAB-BSI) cannot be solely attributed to carbapenem resistance [7,8].

In addition to resistance, virulence is another significant factor influencing patient prognosis. *Acinetobacter baumannii* has traditionally been considered a low-virulence opportunistic pathogen [9]. However, in recent years, the emergence of hypervirulent *Acinetobacter baumannii* (hv-AB) strains has posed a threat to patient prognosis [10,11]. Several reports by Zhou K, Ou HY, and Jones CL et al. have highlighted the identification and outbreak of hv-AB in various hospitals in China and the United States [12,13,14]. Zhou K et al. further observed that patients infected with ST457 hv-AB had a significantly higher 7-day mortality rate compared to those infected with other sequence types (STs) of CC92 (44.4% vs. 14.3%, *p* = 0.01), except for ST195/ST208 [12].

Concerningly, cases of BSI caused by hv-CRAB (hv-CRAB-BSI) have been reported recently. Li J et al. reported a high 21-day mortality rate of 71.4% among patients with hv-CRAB-BSI in China’s central and southern regions [9]. The presence of this pathogen poses a significant threat to public health due to its multidrug resistance and hypervirulence. Previous studies have reported several virulence-related factors in BSI caused by AB, such as capsular polysaccharides [15], type VI secretion systems (T6SS) [16], and biofilm formation [9]. However, current research on hv-CRAB-BSI has mainly concentrated on its resistance and risk factors for mortality, lacking comparative studies on virulence characteristics [17,18,19]. Additionally, the combination use of different antibiotics has been shown to improve treatment outcomes and reduce mortality rates of hv-CRAB-BSI patients [20,21]. However, the optimal regimen for a combination therapy remains unclear, and different strains may exhibit varying responses to different drug combinations. Therefore, it is necessary to evaluate the effective combination therapy for hv-CRAB-BSI in vitro.

This study aimed to compare the clinical and molecular characteristics, as well as the in vitro combination therapy, between hv-CRAB-BSI and non-hv-CRAB-BSI. These findings will contribute to a better understanding of the pathogenesis of hv-CRAB-BSI, improving patient prognosis, and developing rational treatment strategies.

## 2. Results

### 2.1. General Characteristics of CRAB-BSI

A total of 31 strains of CRAB-BSI from adult hospitalized patients were included in this study. The mean baseline age was 63.52 years, with males accounting for 80.6% (*n* = 25). The proportion of patients admitted to the ICU was 90.3% (*n* = 28). The most common underlying disease was hypertension (*n* = 14, 45.2%). The most frequently performed invasive procedure was endotracheal intubation or tracheostomy (*n* = 22, 71.0%). Sepsis was present in 64.5% (*n* = 20) of patients. The overall mortality of patients was 54.8% (*n* = 17) (Table 1).

### 2.2. Grouping of CRAB-BSI and ST Type

**Figure 1:**
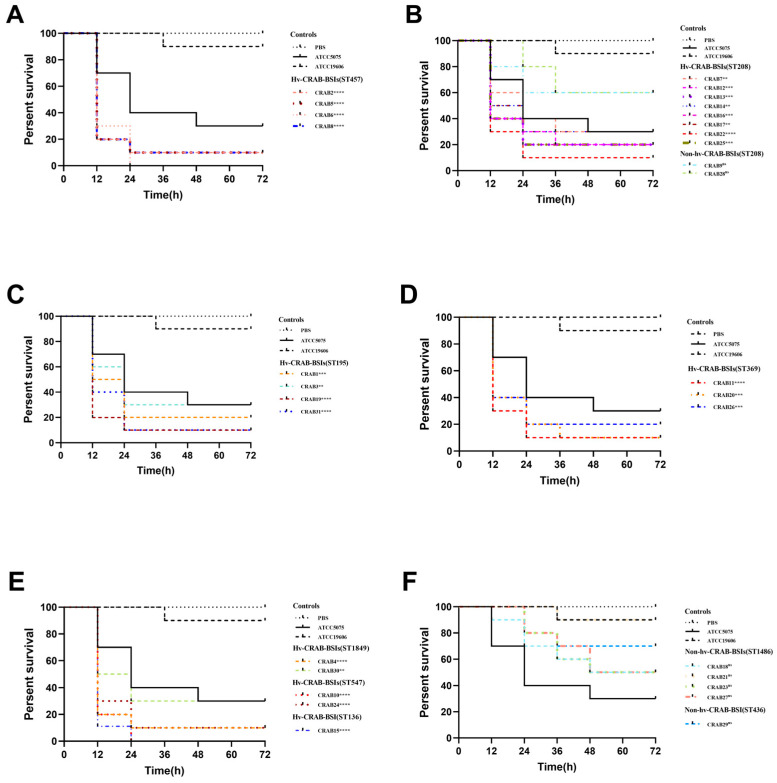
Survival curve illustrating the virulence of 31 cases of CRAB-BSI and the control groups in *Galleria mellonella*. Hypervirulent *Acinetobacter baumannii* strain AB5075 [22] was used as hypervirulent control, while ATCC 19606 and PBS were used as non-hypervirulent control and puncture control, respectively. The log-rank (Mantel–Cox) test was employed to compare the survival curves between the experimental strains and ATCC 19606, ^ns^ not significant; ** *p* < 0.005; *** *p* < 0.001; **** *p* < 0.0001. (**A**) Survival curve of the ST457 CRAB-BSI. (**B**) Survival curve of the ST208 CRAB-BSI. (**C**) Survival curve of the ST195 CRAB-BSI. (**D**) Survival curve of the ST369 CRAB-BSI. (**E**) Survival curve of the ST1849, ST547and ST136 CRAB-BSI. (**F**) Survival curve of the ST1486 and ST436 CRAB-BSI.

The virulence of 31 CRAB-BSI strains was determined using the *Galleria mellonella* infection model, and the strains were categorized accordingly. The results showed that the survival rates of the larvae infected with the non-hypervirulent control strain ATCC 19606 and the hypervirulent control strain AB5075 at 72 h were 90% and 30%, respectively. According to the criteria for hv-CRAB-BSI, the survival curves of 24 strains were significantly lower compared to that of ATCC 19606 (*p* < 0.05) and were either comparable to or lower than that of AB5075, thereby defining these strains as hv-CRAB-BSI. Conversely, 7 strains were classified as non-hv-CRAB-BSI, based on the established criteria (Figure 1 and Table S1).

Both hv-CRAB-BSI and non-hv-CRAB-BSI exhibit a relatively dispersed distribution in multi-locus sequence typing (MLST) profiles. Among the 24 hv-CRAB-BSI strains, they were distributed across 7 different ST types, with ST208 being the most common (*n* = 8, 33.3%), followed by ST457 (*n* = 4, 16.7%), ST195 (*n* = 4, 16.7%), ST369 (*n* = 3, 12.5%), ST547 (*n* = 2, 8.3%), ST1849 (*n* = 2, 8.3%), and ST136 (*n* = 1, 4.2%). Among the 7 non-hv-CRAB-BSI strains, they were distributed across 3 different ST types, with ST1486 being the most common (*n* = 4, 57.1%), followed by ST208 (*n* = 2, 28.6%), and ST436 (*n* = 1, 14.3%) (Figure 1 and Table S1).

### 2.3. Differences in Clinical Characteristics between Hv-CRAB-BSI and Non-Hv-CRAB-BSI

In comparison to patients infected with non-hv-CRAB-BSI, there was a higher prevalence of septic shock development (79.2% vs. 14.3%, *p* = 0.004) and significantly higher mortality rates (66.7% vs. 14.3%, *p* = 0.028) among patients infected with hv-CRAB-BSI. Overall, both groups had a high rate of ICU admission, but there was no significant difference between the two groups (95.8% vs. 71.4%, *p* = 0.120). Regarding age and gender composition, both groups were predominantly composed of elderly males, with no significant difference observed (*p* > 0.05). Regarding underlying comorbidities, both groups had a high prevalence of hypertension, diabetes, coronary heart disease, and other underlying conditions. However, no significant difference was observed between the two groups (*p* > 0.05). Additionally, the probability of invasive operations such as central venous intubation, endotracheal intubation, and tracheotomy was also both high in the two groups (*p* > 0.05) (Table 1).

### 2.4. Differences in Toxome between Hv-CRAB-BSI and Non-Hv-CRAB-BSI

**Figure 2:**
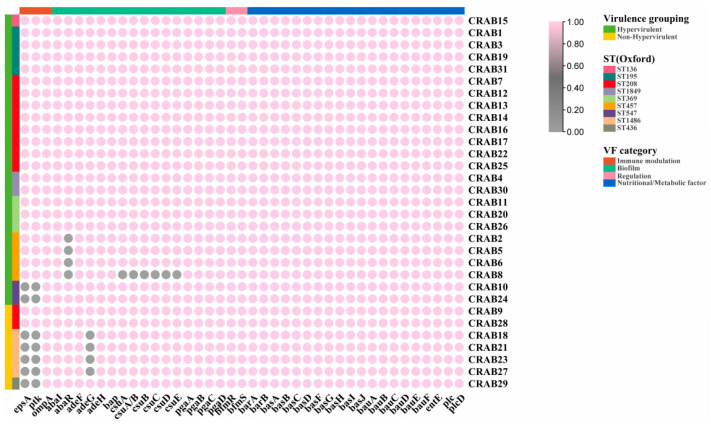
Toxome analysis of hv-CRAB-BSI and non-hv-CRAB-BSI. The genomes were comprehensively compared to the data in the Virulence Factor Database (VFDB), with genes that are present indicated in pink and genes that are absent depicted in grey.

As shown in Figure 2, all 31 CRAB-BSI strains carried genes related to nutrition/metabolism (*barAB*, *basABCDFGHIJ*, *bauABCDEF*, *entE*, *plc*, *plcD*), biofilm formation (*abaI*, *adeF*/*H*, *bap*, *pgaABCD*), regulatory (*bfmRS*), and immune modulation (*ompA*). Notably, there was a significant difference in the detection rates of *ptk* and *epsA* between the two groups (91.7% vs. 28.6%, *p* = 0.002). Among the 24 hv-CRAB-BSI strains, only 2 ST547 strains did not have immune modulation genes *ptk* and *epsA*, while among the 7 non-hv-CRAB-BSI strains, only 2 ST208 strains had *ptk* and *epsA*. Furthermore, *abaR* related to biofilm formation was not detected in 4 strains of ST457 hv-CRAB-BSI, and *adeG* related to biofilm formation was not detected in 4 strains of ST1486 non-hv-CRAB-BSI.

### 2.5. Differences in Resistome between Hv-CRAB-BSI and Non-Hv-CRAB-BSI

**Figure 3:**
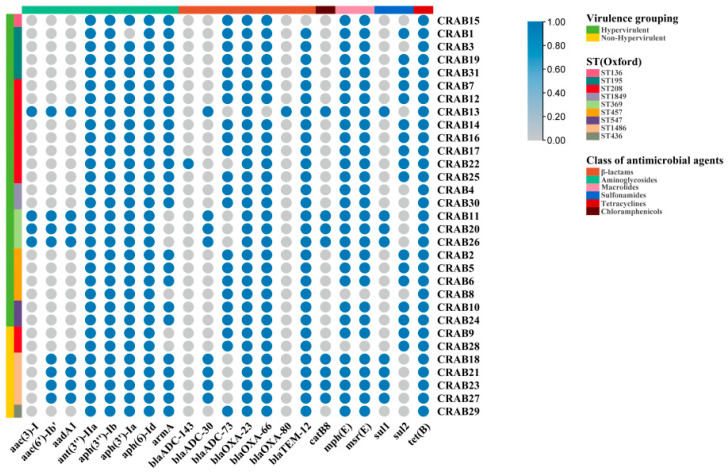
Resistome analysis of hv-CRAB-BSI and non-hv-CRAB-BSI. The genomes were comprehensively compared to the data in NCBI database of ABRicate v.1.0.1, with genes that are present indicated in blue and genes that are absent depicted in grey.

To analyze the relationship between virulence and antimicrobial resistance, we compared the differences in antimicrobial-resistance genes between the two groups. Among the 31 CRAB-BSI strains, the most prevalent carbapenem-hydrolyzing class D β-lactamases (CHDLs) were distributed as follows: *bla*_OXA-23_, 100%; OXA-51-like, including *bla*_OXA-66_ and *bla*_OXA-80_, 100%. None of the strains carried genes for class B metallo-β-lactamases (MBL) (*bla*_VIM_, *bla*_IMP_, and *bla*_NDM_), as well as the gene encoding KPC-type Class A carbapenemases (Figure 3). In addition, other resistance genes were detected at rates above 80%, including β-lactamase resistance genes (*bla*_ADC-73_, *bla*_TEM-12_), aminoglycoside resistance genes (*ant(3″)-IIa*, *aph(6)-Id*, *aph(3″)-IIa*, *aph(3″)-Ib*, *aph(3′)-Ia*), macrolide resistance genes (*msr(E)*, *mph(E)*), tigecycline resistance gene *tet(B)*, and sulfonamide resistance gene *sul2*. Interestingly, the difference in resistance genes between ST369 hv-CRAB-BSI and ST1486 non-hv-CRAB-BSI was only observed in gentamicin resistance genes *aac (3)-I* and *armA*. The former detected *aac (3)-I* but not *armA*, while the latter detected *armA* but not *aac (3)-I* (Figure 3).

### 2.6. Differences in Antimicrobial Susceptibility and Synergistic Effect between Hv-CRAB-BSI and Non-Hv-CRAB-BSI

As shown in Table 2, the overall resistance rate of the 31 strains of CRAB-BSI to colistin (COL) was 0%, while the resistance rates to tigecycline (TIG) and minocycline (MIN) were 25.8% and 64.5%, respectively. The resistance rates for other antimicrobial agents, such as imipenem, ceftazidime, ampicillin/sulbactam, tobramycin, and levofloxacin were higher than 77.4%. Notably, compared to non-hv-CRAB-BSI, MIN exhibited stronger sensitivity against hv-CRAB-BSI (45.8% VS. 0%, *p* = 0.03), while gentamycin showed weaker sensitivity against hv-CRAB-BSI (0% VS. 28.6%, *p* = 0.045). There were no significant differences in the sensitivity of the other antimicrobial agents between the two groups (*p* > 0.05).

Considering the nephrotoxicity of COL, combination antimicrobial therapy may be necessary [23]. We evaluated six combinations based on COL and TIG to explore effective treatment options for hv-CRAB-BSI. These combinations were COL+MIN, COL+ meropenem (MER), COL+ cefoperazone/sulbactam (CFS), TIG+COL, TIG+MER, and TIG+CFS. We found that synergism was found in four, one, two, zero, one, zero strains in combination of COL+MIN, COL+MER, COL+CFS, TIG+COL, TIG+MER, and TIG+CFS, respectively. There were no significant differences in the effectiveness of these six combination regimens between the two groups (*p* > 0.05). However, for all CRAB-BSI strains, the combination of COL+MIN showed the best efficacy. The proportions of synergism and additive effects for the combinations of COL+MIN, COL+MER, COL+CFS, TIG+COL, TIG+MER, and TIG+CFS were 100%, 96.8%, 58.1%, 67.7%, 74.2%, and 48.4%, respectively (Tables S2 and S3).

## 3. Discussion

*Acinetobacter baumannii*, due to its multi-drug resistance and virulence, has become one of the most concerning species worldwide [24]. Bloodstream infections (BSI) caused by CRAB were increasingly prevalent among hospitalized patients [17,25,26,27]. The high mortality rate associated with such infections is alarming. Analyzing the molecular characteristics of such pathogens and exploring effective treatment strategies is crucial. In this study, using the *Galleria mellonella* infection model, we compared the differences in the clinical and molecular characteristics, as well as combination therapy in vitro between hv-CRAB-BSI and non-hv-CRAB-BSI.

In our study, the results of the virulence grouping of strains by the *Galleria mellonella* infection model were consistent with the clinical outcome of patients. Patients with hv-CRAB-BSI were more likely to develop septic shock (79.2% VS. 14.3%, *p* = 0.004) and the mortality rate was significantly higher than that of non-hv-CRAB-BSI (66.7% VS. 14.3%, *p* = 0.028). Through a comparative genomic analysis of virulence genes, we found that only two genes, *epsA* and *ptk*, showed significant differences between hv-CRAB-BSI and non-hv-CRAB-BSI (91.7% VS. 28.6%, *p* = 0.002). The *epsA* gene encodes a putative polysaccharide export outer membrane protein (EpsA), while *ptk* encodes a putative protein tyrosine kinase (PTK) [28]. EpsA and PTK have been found to promote the synthesis of K1 capsular polysaccharide, leading to increased virulence and helping the bacterial strains evade the host’s innate immune system. This enables them to survive longer in human ascites, human serum, and mouse soft tissue infection models, while also indirectly influencing the motility of the strains [28]. Additionally, PTK and EpsA are critical to the *Acinetobacter baumannii* capsular-positive phenotype and do not share any significant homology with human proteins. These two conserved proteins can be used as potential targets for anti-virulence drugs [29]. Our study further reveals the correlation between the virulence genes *epsA* and *ptk* and the increased virulence of *Acinetobacter baumannii*, which will provide evidence for the successful development of such anti-virulence drugs.

Notably, we also found that all ST457 hv-CRAB-BSI strains lacked the *abaR* gene. The *abaI*/*abaR* QS quorum sensing system was found to be prevalent in clinical strains of *Acinetobacter baumannii* and played a crucial role in surface-associated motility [30]. Sun X et al. found that the pathogenicity of the *abaR* mutant was significantly higher than that of the wild strain in the *Galleria mellonella* infection model and the mouse infection model, suggesting that the absence of *abaR* enhanced the cytotoxicity of the strain and helped it evade host immunity [31]. In our study, all four CRAB-BSI strains lacking *abaR* belonged to the ST457 type, and the outcome of patients infected with these strains was death, with a survival rate of ≤10% for the larvae infected with these strains. Interestingly, our research group’s previous study also found that *abaR* was absent in hypervirulent ST457 *Acinetobacter baumannii* [12]. Furthermore, Li J et al. reported that 4 ST457 hv-CRAB strains also lacked *abaR*, and three out of four patients infected with these strains died, with a 5-day survival rate of ≤20% for infected larvae [9]. Therefore, we speculate that the loss of the *abaR* gene is one of the main reasons for the enhanced virulence of ST457 *Acinetobacter baumannii*. In addition, we also found that all ST1486 non-hv-CRAB-BSI lacked *adeG*, which encodes the AdeFGH efflux pump. The amount of biofilm formation of *Acinetobacter baumannii* increased with the increase of *adeG* expression [32]. Further research should focus on the development of anti-virulence drugs targeting *abaR* and *adeG*.

In this study, all strains were sensitive to COL. However, considering the nephrotoxicity of COL, combination therapy may be necessary. We conducted checkerboard broth microdilution to explore suitable combination therapy regimens. Unfortunately, although the resistance rate of hv-CRAB-BSI to MIN alone was lower compared to non-hv-CRAB-BSI (54.2% VS. 100%, *p* = 0.03), we did not observe any significant differences between the two groups in these selected combinations. Furthermore, synergism was rarely observed in these combinations. Overall, the combination of COL+MIN showed the best efficacy against CRAB-BSI, demonstrating a synergistic or additive effect. This finding was consistent with the study by Qu X et al., who demonstrated the potential effectiveness of the combination of polymyxin B and MIN for treating CRAB-BSI using a PK/PD model in vitro [33]. In addition, several studies have reported the effectiveness of MIN alone or in combination in the treatment of CRAB [34,35,36,37]. Notably, the combination of COL+MER also showed good efficacy in our study, although synergism was observed in only one strain when combined with COL+MER. Meanwhile, the results of the largest randomized controlled trial to date did not support the use of COL+MER combination therapy for treating CRAB infections [38]. Therefore, there is still a lot of controversy about whether to use the combination of COL+MER to treat CRAB-BSI. Meanwhile, we also found that the overall effect of TIG-based combination therapy regimens was poor. Several studies have reported that clinicians need to use TIG cautiously when the MIC of CRAB was greater than 2 mg/L [39,40]. Interestingly, in our study, the MICs of TIG for all strains were greater than 2 mg/L, and the MIC_50_ and MIC_90_ were 4 mg/L and 8 mg/L, respectively. Therefore, clinicians should be cautious in using TIG-based combination therapy regimens when dealing with patients infected by such strains.

Our study also has the following limitations. First, the sample size was small. Although there were significant differences in the susceptibility of MIN and gentamicin against hv-CRAB-BSI and non-hv-CRAB-BSI, these findings need to be further validated with larger sample sizes and larger multicenter studies. Additionally, considering the heterogeneity of clinical backgrounds, although we found a good correlation between clinical outcome and the survival time of infected larvae, we should not simply attribute patient deaths to bacterial virulence. Further research is necessary to accurately determine the role of bacterial virulence in patients with CRAB-BSI.

## 4. Materials and Methods

### 4.1. Strains and Ethics

A total of 31 CRAB-BSI strains from hospitalized patients of The First Affiliated Hospital of Guangzhou Medical University were included. The baseline information of patients such as clinical outcomes were collected. The species of all the strains were identified by MALDI-TOF-MS (Bio Mérieux, Craponne, France) and confirmed by 16S rDNA sequencing and whole-genome sequencing. The study was approved by the Research Ethics Committee of The First Affiliated Hospital of Guangzhou Medical University (ES-2023-057-01). Informed consent was waived by the committee.

### 4.2. Galleria mellonella Infection Model and Grouping

The *Galleria mellonella* infection model was constructed following previously described methods [41,42], with minor adjustments. Healthy *Galleria mellonella* larvae weighing 300 mg were selected for the assays. The strains, including 31 CRAB-BSIs, ATTCC 19606, and AB5075, were cultured overnight in a shaker (37 °C, 200 rpm) (Labnet, Edison, NJ, USA). The overnight cultures were then centrifuged (5 min, 6000 rpm) to remove the supernatant and washed once with phosphate-buffered saline (PBS). A 20 µL suspension of PBS containing 1.5 × 10^7^ colony-forming units (cfu) was injected into the last left proleg of each larva. The injected larvae were incubated at 37 °C for 72 h. Larval death was defined as the failure to respond to physical stimuli and the blackening of the body’s surface. The death status of the larvae was assessed every 12 h. Each experiment included three control groups: larvae injected with 20 µL PBS as the control for a puncture, ATCC 19606 as a non-hypervirulent control strain, and AB5075 [22] as a hypervirulent control strain. Each experiment was repeated three times, and 10 larvae were injected into each group.

Survival curves were generated using GraphPad Prism 9.0 software, and a log-rank (Mantel-Cox) test was used to evaluate differences in survival. To be designated as a high-virulence strain (hv-CRAB-BSI), CRAB-BSI must meet both of the following criteria: (1) The survival curve of larvae infected with CRAB-BSI must be significantly lower than that of larvae infected with the non-high-virulence control strain ATCC 19606 (*p* < 0.05). (2) The survival curve of larvae infected with CRAB-BSI must either be significantly lower (*p* < 0.05) or not significantly different (*p* ≥ 0.05) from that of larvae infected with the high-virulence control strain AB5075. All other strains were classified as non-high-virulence strains (non-hv-CRAB-BSI).

### 4.3. Whole-Genome Sequencing (WGS) and Bioinformatic Analysis

For the 31 CRAB-BSI strains, whole-genome sequencing (WGS) analysis was conducted. Monoclones of bacteria were selected from an agar plate and cultured in 4 mL of LB broth at 37 °C for 16 h (200 rpm/min). Genomic DNA was extracted using the Bacterial DNA Kit D3350 (Omega Bio-Tek, Norcross, GA, USA). WGS was performed by Novogene (Beijing, China) using the Illumina Novaseq 6000 platform (Illumina, San Diego, CA, USA). The clean data were trimmed and assembled using Shovill [43]. The assembled contigs were then annotated using Prokka 1.14.6 [44]. Antibiotic resistance genes and virulence genes were detected using the NCBI database and VFDB database in ABRicate v.1.0.1, respectively [45,46]. The draft genomes were uploaded to the PubMLST server (https://pubmlst.org/abaumannii/ (accessed on 26 November 2023)) for assigning sequence types (STs) according to the Oxford schemes [47].

### 4.4. Antimicrobial Susceptibility Testing

The broth microdilution method was used to determine the minimum inhibitory concentrations (MICs) of antimicrobial agents. The tested antimicrobial agents include carbapenems (imipenem and meropenem (MEM)), cephalosporins (ceftriaxone, ceftazidime, cefepime, and cefoperazone/sulbactam (CFS)), aminoglycosides (amikacin, gentamicin, and tobramycin), tetracyclines (minocycline (MIN) and tigecycline (TIG)), penicillin/β-lactamase inhibitor complexes (ampicillin/sulbactam and piperacillin/tazobactam), fluoroquinolones (ciprofloxacin and levofloxacin), colistin (COL), and trimethoprim/sulfamethoxazole. These antimicrobial agents were purchased from Macklin (Shanghai, China), except for colistin which was acquired from Sigma-Aldrich (Saint Louis, MO, USA). The results of TIG were interpreted based on the clinical breakpoints recommended by the US Food and Drug Administration (FDA) [48]. The results of other antibiotics were interpreted according to the clinical breakpoints recommended by the Clinical Laboratory and Standards Institute (CLSI) [49]. *Pseudomonas aeruginosa* ATCC 27853 and *Escherichia coli* ATCC 25922 were used as quality control strains.

### 4.5. Checkerboard Broth Microdilution

For the evaluation of combination effects, a checkerboard broth microdilution method was performed for the following antibiotic combinations: COL+MEM, COL+MIN, COL+CFS, TIG+COL, TIG+MEM, and TIG+CFS. Briefly, based on the single-agent MIC results of the strain, a gradient range of drugs A and B in combination was designed. A 96-well microtiter plate was prepared, with each well containing 25 μL of drugs A and B at different concentrations. A volume of 50 μL of a diluted bacterial suspension (1.5 × 10^6^ CFU/mL) was added to the plate, followed by incubation at 37 °C for 18 h. The fractional inhibitory concentration index (FICI) was calculated for each combination using the formula: FICI = (MIC of A in combination/MIC of A alone) + (MIC of B in combination/MIC of B alone). The interpretation of FICI results for each combination is as follows: FICI ≤ 0.5, synergism; 0.5 < FICI ≤ 1, additive; 1 < FICI ≤ 4, indifference; FICI > 4, antagonism [50].

### 4.6. Statistical Analysis

Statistical analysis was conducted using SPSS 25.0 software. Measurement data were presented as mean ± standard deviation (X ± S) while counting data were expressed as a percentage (%). The differences between rates were assessed using χ2 or Fisher’s exact tests, as appropriate. The survival curve of *Galleria mellonella* was analyzed using the Kaplan-Meier method, and the significance of survival curve differences was determined using the Log-rank test. A *p* value of <0.05 was considered statistically significant.

## 5. Conclusions

In summary, we found that patients infected with hv-CRAB-BSI had a significantly higher mortality rate compared to non-hv-CRAB-BSI. EpsA and PTKmay serve as potential drug targets for combating the virulence of CRAB-BSI. The *abaR* gene may play a crucial role in determining the virulence of ST457 CRAB-BSI. Although synergism was rarely observed in these selected combinations, the combination of COL+MIN showed the best efficacy for treating CRAB-BSI, regardless of whether the infections were hv-CRAB-BSI or non-hv-CRAB-BSI. These findings highlight the importance of analyzing molecular characteristics and exploring effective treatment strategies for hv-CRAB-BSI.

## Figures and Tables

**Table 1:** Clinical baseline characteristics of 31 patients infected with CRAB-BSI.

Variables	Total (*n* = 31)	Groups		*p*-Value
		Hv-CRAB-BSI (*n* = 24)	Non-hv-CRAB-BSI (*n* = 7)	
Age (yr, X ± S)	63.5 ± 20.7	63.7 ± 20.4	63.0 ± 23.3	0.94
Sex at birth, Male	25 (80.6%)	20 (83.3%)	5 (71.4%)	0.59
ICU admission	28 (90.3%)	23 (95.8%)	5 (71.4%)	0.12
Combined underlying disease	21 (67.7%)	15 (62.5%)	6 (85.7%)	0.379
Hypertension	14 (45.2%)	11 (45.8%)	3 (42.9%)	1
Diabetes	7 (22.6%)	6 (25.0%)	1 (14.3%)	1
Coronary artery disease	12 (38.7%)	9 (37.5%)	3 (42.9%)	1
Cerebral hemorrhage	6 (19.4%)	6 (25.0%)	0 (0.0%)	0.293
COPD ^#^	3 (9.7%)	3 (12.5%)	0 (0.0%)	1
Invasive operation	24 (77.4%)	20 (83.3%)	4 (57.1%)	0.302
Peripherally inserted central catheter	20 (64.5%)	17 (70.8%)	3 (42.9%)	0.21
Tracheal intubation and tracheotomy	22 (71.0%)	18 (75.0%)	4 (57.1%)	0.384
Catheter	20 (64.5%)	18 (75.0%)	2 (28.6%)	0.067
Gastric intubation	20 (64.5%)	18 (75.0%)	2 (28.6%)	0.067
Thoracic and abdominal drainage tube	8 (25.8%)	8 (33.3%)	0 (0.0%)	0.146
Septic shock	20 (64.5%)	19 (79.2%)	1 (14.3%)	**0.004**
Clinical outcome				
Death	17 (54.8%)	16 (66.7%)	1 (14.3%)	**0.028**
Recover	14 (45.2%)	8 (33.3%)	6 (85.7%)	

^#^ COPD: Chronic obstructive pulmonary disease; The bolded parts indicate statistical significance (*p* < 0.05).

**Table 2:** Antimicrobial susceptibility of 31 CRAB-BSI strains.

Antimicrobial Agents	Resistance Rate (%)		*p*-Value *	Resistance Rate (%)	MIC_50_ (mg/L)	MIC_90_ (mg/L)
	Hv-CRAB-BSI (*n* = 24)	Non-hv-CRAB-BSI (*n* = 7)		Total (*n* = 31)		
Imipenem	24 (100)	7 (100)	-	31 (100)	64	128
Meropenem	24 (100)	7 (100)	-	31 (100)	64	128
Ceftriaxone	24 (100)	7 (100)	-	31 (100)	128	128
Ceftazidime	24 (100)	7 (100)	-	31 (100)	128	128
Cefepime	24 (100)	7 (100)	-	31 (100)	128	128
Cefoperazone/sulbactam	24 (100)	6 (85.7)	0.226	30 (96.7)	128	128
Tigecycline	5 (20.8)	3 (42.9)	0.335	8 (25.8)	4	8
Minocycline	13 (54.2)	7 (100)	**0.033**	20 (64.5)	16	32
Colistin	0 (0)	0 (0)	-	0 (0)	0.5	1
Ampicillin/sulbactam	24 (100)	7 (100)	-	31 (100)	128/64	128/64
Piperacillin/tazobactam	24 (100)	7 (100)	-	31 (100)	128/4	128/4
Levofloxacin	24 (100)	7 (100)	-	31 (100)	16	64
Ciprofloxacin	24 (100)	7 (100)	-	31 (100)	8	8
Gentamycin	24 (100)	5 (71.4)	**0.045**	29 (93.5)	64	64
Amikacin	20 (83.3)	5 (71.4)	0.596	25 (80.6)	128	128
Tobramycin	17 (70.8)	7 (100)	0.161	24 (77.4)	16	64
Trimethoprim/sulfamethoxazole	24 (100)	7 (100)	-	31 (100)	16/304	32/608

*: The bolded parts indicate statistical significance (*p* < 0.05).

## Data Availability

All sequences of the strains included in this study are available under BioProject accession number PRJNA1083246.

## Supplementary Materials

The following supporting information can be downloaded at: https://www.mdpi.com/article/10.3390/antibiotics13090807/s1, Table S1: Grouping of 31 CRAB-BSI based on infection outcomes of *Galleria mellonella* larvae; Table S2: Combinatory antibacterial activity of different antibiotic combinations on 31 CRAB-BSIs. Table S3: The proportion of synergism and additive in different antibiotics combinations to 31 CRAB-BSI strains.
