# Supplementary material for: Comparison of Hypervirulent and Non-Hypervirulent Carbapenem-Resistant Acinetobacter baumannii Isolated from Bloodstream Infections: Mortality, Potential Virulence Factors, and Combination Therapy In Vitro
Source: Antibiotics (Basel). 2024 Aug 26;13(9):807. doi: 10.3390/antibiotics13090807 (PMC11428969; doi:10.3390/antibiotics13090807)
Supplement: Supplementary file 1 [file antibiotics-13-00807-s001.zip › Table S1.pdf]

Table S1. Grouping of 31 CRAB-BSI based on infection outcomes of Galleria mellonella larvae

Grouping of CRAB-BSI	Isolates	ST types	P value†		Clinical outcomes
			P1(AB5075)	P2(ATCC19606)	
Puncture control	PBS	NA	NA	NA	NA
Hypervirulent control	AB5075	NA	NA	0.0052	NA
Non-hypervirulent control	ATCC19606	NA	0.0052	NA	NA
Hv-CRAB-BSI	CRAB1	ST195	0.4398	0.0009	death
	CRAB2	ST457	0.0798	<0.0001	death
	CRAB3	ST195	0.8425	0.0043	death
	CRAB4	ST1849	0.0798	<0.0001	death
	CRAB5	ST457	0.0798	<0.0001	death
	CRAB6	ST457	0.0195*	<0.0001	death
	CRAB7	ST208	0.8681	0.0043	recover
	CRAB8	ST457	0.0798	<0.0001	death
	CRAB10	ST547	0.0101*	<0.0001	death
	CRAB11	ST369	0.0698	<0.0001	death
	CRAB12	ST208	0.4091	0.0009	death
	CRAB13	ST208	0.3653	0.0008	death
	CRAB14	ST208	0.7466	0.0041	recover
	CRAB15	ST136	0.0101*	<0.0001	death
	CRAB16	ST208	0.3653	0.0008	recover
	CRAB17	ST208	0.7466	0.0041	recover
	CRAB19	ST195	0.0798	<0.0001	death
	CRAB20	ST369	0.1636	0.0001	recover
	CRAB22	ST208	0.1090	<0.0001	death
	CRAB24	ST547	0.1090	<0.0001	recover
	CRAB25	ST208	0.3653	0.0008	recover
	CRAB26	ST369	0.3653	0.0008	death
	CRAB30	ST1849	0.7466	0.0041	recover
	CRAB31	ST195	0.1492	0.0001	death
Non-hv-CRAB-BSI	CRAB9	ST208	0.2530	0.1089	recover
	CRAB18	ST1486	0.2874	0.0502	death
	CRAB21	ST1486	0.0052**	>0.9999	recover
	CRAB23	ST1486	0.2034	0.0526	recover
	CRAB27	ST1486	0.1889	0.0586	recover
	CRAB28	ST208	0.0756	0.1101	recover
	CRAB29	ST436	0.0815	0.2450	recover

*:The mortality rate of the larvae infected with this bacterium was significantly higher than that of AB5075 within 72 hours. **:The mortality rate of the larvae infected with this bacterium was significantly lower than that of AB5075 within 72 hours. †:The log-rank test was employed to assess the differences of survival curves between the experimental strains and the control strain(AB5075 and ATCC19606). A p-value of <0.05 was considered statistically significant.
