# Supplementary material for: Comparison of Hypervirulent and Non-Hypervirulent Carbapenem-Resistant Acinetobacter baumannii Isolated from Bloodstream Infections: Mortality, Potential Virulence Factors, and Combination Therapy In Vitro
Source: Antibiotics (Basel). 2024 Aug 26;13(9):807. doi: 10.3390/antibiotics13090807 (PMC11428969; doi:10.3390/antibiotics13090807)
Supplement: Supplementary file 1 [file antibiotics-13-00807-s001.zip › Table S3.pdf]

Table S3 The proportion of synergism and additive in different antibiotics combinations to 31 CRAB-BSI strains

Antibiotics combinations	Proportion of synergism and additive（%）			P value
	Total（n=31）	Hv-CRAB-BSI（n=24）	Non-hv-CRAB-BSI（n=7）	
COL+CFS	18（58.1%）	14（58.3%）	4（57.1%）	1.00
COL+MER	30（96.8%）	24（100.0%）	6（85.7%）	0.226
COL+MIN	31（100%）	24（100.0%）	7（100.0%）	-
TIG+COL	21（67.7%）	15（62.5%）	6（85.7%）	0.379
TIG+MER	23（74.2%）	17（70.8%）	6（85.7%）	0.642
TIG+CFS	15（48.4%）	10（41.7%）	5（71.4%）	0.220

MER, Meropenem; COL, Colistin; TIG, Tigecycline; MIN, Minocycline; CFS, Cefoperazone/sulbactam;

The P value of <0.05 was considered as statistically significant.
